# Practical Medicine and Pathology

**Published:** 1875-10

**Authors:** 


### III. Practical Medicine and Pathology.

1. *Differential Diagnosis of Instestinal Invagination.* (*La France Mèdicale*, No. 47, 1875.)

In an article of the "*Archiv. fur Prakt. Heilkunde*," Dr. O. Lachtenstein gives a certain number of symptoms serving to differentiate intestinal intussusception of the large intestine from that of the small one:

1st. Intestinal invagination is rare in the first year of life, and, in fact, in the whole of infancy.

2d. In adults the progress of the invagination of the ileum is more rapid, the phenomena are more marked than in ileo-cæcal invagination, and that of the colon.

Chronicity is rare in invaginations of the small instestine; it is more frequent in the ileo-cæcal portion, or the colon. Grave symptoms of collapse become manifest more frequently at the beginning of this affection.

3d. *Muco-sanguinolent discharges* are the rule in all invaginations, wherever their seat may be. The author has observed dejections of fæcal material of an aspect wholly normal after a diarrhœa, in ileo-cæcal invaginations; he has seen it only once, in an adult, in invagination of the colon.

4th. *Meteorism* is a very variable symptom; usually it is wanting in ileo-cæcal invaginations. In invaginations of the descending colon, it is generally seen to occupy the transverse colon; afterwards it extends over the whole abdomen. In invagination of the ileum, it is found occupying a limited area, especially the central part of the abdomen, without invading the lateral or epigastric regions.

5th. *Tenesmus* is rare in invagination of the ileum; it is frequent in that of the colon and the ileo-cæcum.

6th. In invagination of the ileum no *tumor* ordinarily exists, or else (if there be one) it is seated in the centre of the hypogastrium; when it is seated in the cæcal region, especially if it be stationary for some time, it shows an invagination of the ileum or the ileo-cæcum. The extension of the tumor, when it appears suddenly and corresponds to the course of the colon, indicates most strongly an ileo-cæcal invagination; less so, one of the colon; and excludes invagination of the ileum. The situation of the tumor in the left lateral region of the abdomen indicates an invagination of the ileo-cæcum or of the colon. We can never feel the tumor in the rectum, and prolapsus of it neverappears in invagination of the ileum without complications. Changes in the consistence, the appearance and disappearance of the tumor, are seen particularly in invagination of the ileo-cæcum.

2. *On the Toxic Principle of Putrid Blood.* FELTZ. (*Centralblatt für Chirurgie.*)

V. Feltz had putrid blood exposed to the air for several months; and on examining with the microscope from time to time he found that the vibriones and germs gradually were losing their active mobility and finally disappeared entirely. At the same time the odor became less pungent on account of the decrease of ammonia productions. He injected from one-half to two cubic centimetres of such blood into the crural veins of six dogs, and all exhibited the signs of a putrid infection: increase of temperature, loss of appetite, vomiting and diarrhœa; four of the dogs died after ten or twelve days; two survived and slowly recovered. The same result was obtained from an injection of blood which had stood in the sun during five months, then was perfectly dried, pulverized and mixed with distilled water. And the blood of all the dogs thus treated was teeming with vibriones and showed all the changes and the disintegration of the red corpuscles so characteristic of a putrid infection. Feltz then comes to the conclusion that the dry putrid blood contains germs which, motionless and apparently dead, revive when introduced into normal blood and produce the signs of a septic infection.

3. *Intestinal Diseases treated by introducing into the Intestinal Tract large quantities of Fluid.* MOSLER. (*Memorabilien*, July, 1875.)

Prof. Mosler (Greifswald) has largely experimented on animals and men on the effect of copious injections into the colon. By the pressure of a fountain syringe he has introduced from one to five quarts of warm water, and has never seen any ill effect from it if only the water is introduced slowly and gradually; while the patient is lying on his back the water thus injected is forced up as far as the ileo-cæcal valve and sometimes even beyond it into the ileum. The professor considers these copious injections as the best means of effectually cleansing the mucous membrane of the bowels from irritating substances and of applying disinfectants and astringent remedies over a larger surface of the mucous lining. During the summer of last year he obtained very favorable results from their use in cholera infantum. Even the smallest infants while held on the lap of a nurse would bear them well. As disinfectants, permanganate of potassa and salicylic acid were used. One and one-half drachms of the salicylic acid were dissolved in two quarts of warm water; and of the permanganate of potassa, he dissolved one drachm in two ounces of water and added two tablespoonsful of this solution to the quart of warm water. This topical treatment proved very gratifying in the various forms of dysenteria; a few "washings" removed the tenesmus and diminished the number of stools. In typhoid fever they seemed to lessen the tympanitis and the frequency of evacuations, and from his comparative observations of similar cases, some of which were treated by copious injections while the others were not, the doctor is inclined to believe that the disease takes a milder course under the local treatment. And the doctor has also convinced himself that the copious injections of warm water greatly insure a complete expulsion of tapeworms which have previously been acted on by the well-known internal medicines. In support of this claim he gives the history of several cases of which we will quote the following: A farmer, aged 23 years, having complained for some time of much headache, drowsiness, weakness and dislike to work, discovered pieces of a tapeworm in his stool on July 5, 1874, and July 7 he was admitted to the hospital. In the afternoon of that day he took castor oil and in the evening his colon was washed with one and one-half quarts of warm water and milk. July 8, at 7 A. M., another dose of castor oil; at 8, 9 and 10 A. M., ten pills of ext. granat. (℞. Ext. Granat. Spirit. ʒ ii, Pulv. Rad. Altheæ, q. s. ut f. pilulæ, No. 30). At 12 and 3 P. M., copious injections; but the water returned without the worm. At 4 P. M., however, the links began to come out, and the nozzle of the tube was then carefully introduced into the anus and one and one-half quarts of tepid water injected, which the patient was enjoined to hold as long as possible. Half an hour later, the water was discharged slowly and with it a coil of tapeworms, that on a closer examination proved to contain three complete specimens of tænia mediocanellata with their heads.

4. *Treatment of Diseases of Respiration and Circulation by the Pneumatic Method.* Dr. A. Rose. (*Med. Record*, Aug. 28, 1875.)

This paper is mostly a description of the experience of European practitioners with the "pneumatic method." This method consists simply in allowing patients to inhale a condensed atmosphere or to expire into a rarefied one, or to inhale a rarefied one, according as the exigencies of the case, brought about by disease of the lungs or heart, may seem to demand in each instance. It is really forcing condensed air into the lungs by reason of its greater pressure—elasticity, thus aiding the inspiratory effort; or it is sucking air out of the chest by the expiration into a rarefied atmosphere, thus aiding the expiratory act; or it is compelling the inspiratory effort to be unusually intense and powerful by making the air inhaled rare.

The treatment has been tried by Waldenburg, Hauke, and others, in emphysema to aid expiration, in that disorder so difficult; in incipient pulmonary tuberculosis where condensed air has been inspired to give the lungs a greater amount of oxygen and to aid the inspiratory effort, and in bronchitis—in all it is claimed with good results.

Several apparatuses are described for the administration of this treatment, that of Biedert seeming to fulfill all requirements best. This machine is made in the form of an accordeon placed in a vertical position and so arranged that one end-board is fixed while the other is movable, and the whole apparatus being suspended upon joints that allow of its being inverted easily. Weights of various sizes are attached to the movable end-piece, which, as will be seen, with the apparatus in one position compress its contained air and in a reverse position, by pulling down, rarefy it.

The condensation or tension of the air used varied from one-sixtieth to one-twentieth of an atmosphere. Patients were required to use the apparatus for a few minutes each day.

Waldenburg says, "inspiration of condensed air, as well as expiration into rarefied air, increase permanently the vital capacity of the lungs (as shown by spirometry), and the power of inspiration and expiration as measurable by the pneumatometer." Some cases he reports, seem fairly to bear him out in this statement.

As in inspiration of condensed air there is increased intra-thoracic pressure, tending to increase the force of the out-going or arterial blood and to retard the motion of the in-coming venous blood, thus increasing the pressure in the periphera of the greater circulation and lessening the pressure within the chest, and decreasing the amount of blood in the lesser circulation, it is argued this expedient may be of value in any cardiac disease where it is desirable to lessen the tension of the heart walls and facilitate the passage of blood through its cavities and through the lesser circulation.

Chronic valvular disease, chronic inflammatory processes in the lungs, and bronchial catarrh of severe forms, are instanced as cases where the treatment would be useful.

Of course inhalation of rarefied air has exactly an opposite effect and the indications for its use would be opposite.

5. *Some of the Conditions under which Pneumonia proves Fatal.* FRANCIS DELAFIELD, M.D. (*Med. Record*, Aug. 21, 1875.)

Statistics are presented of 122 cases of death from pneumonia, where post-mortem examinations were made by or under the direction of the writer. Data are furnished also regarding 1,134 deaths from the disease, in New York, during the year ending March, 1874, and 1,276

during the year following. Comparing the death rate in the last two series of cases with the line of mean temperature for each month of the year, they are found to nearly correspond, the high rate of mortality attending the low temperature. These series of cases were marked by a decided tendency to death on certain days of the disease. "Beginning with a few deaths on the first day of the disease, the curve of mortality runs rapidly up and reaches its maximum on the seventh day, then falling somewhat on the eighth and ninth days, it ascends on the tenth. From the tenth day there is a rapid decrease in the deaths, until the fourteenth day, when the curve rises abruptly. In the same way there is a third maximum on the twenty-first day, and a fourth on the twenty-eighth day." The illustrative charts of the cases respectively of the two years correspond in the rise and fall of the death rate, in a most remarkable manner; the seventh day fatality being marked in both.

Of the 122 cases examined post-mortem, nearly half died at a time when the lungs were passing from the condition of red to that of gray hepatization, while more than a quarter died while in the stage of red hepatization. Comparing the histories of the cases with the autopsies, it appears that in one case red hepatization continued until the eleventh day, and in one case that this stage was completed in 24 hours; also that the disease may be passing from red into gray hepatization by the second day, and it may not have become completely gray by the eighteenth day.

Complete gray hepatization is not reached usually until the seventh day.

Of the 122 cases, in 35, portions of both lungs were involved, and in 25 of these cases not more than one lobe of one lung was left unsolidified.

In 32 cases one entire lung was hepatized—the right in 23 and the left in 9.

In 54 cases only one lobe was hepatized; in 24 cases the upper lobe alone, in 30 cases the lower one.

The right lung alone was involved in 63 cases; the left alone in 23. Complications occurred in all but 15 of the 122 cases.

The complicating lesions occurred as follows: œdema of remaining part of lung in 28 cases; emphysema and chronic bronchitis, 21 cases; a large amount of purulent serum or fibrin in one or both pleural cavities, 16 cases; acute pericarditis, 12 cases; fatty liver, 12 cases; valvular disease of heart, 6 cases; cirrhotic liver, 3 cases; chronic phthisis, gangrene of the lung and basilar meningitis, 2 cases each; waxy liver, 1 case.

"The kidneys were in a condition of chronic Bright's disease in *thirty-four cases.* The kidneys were in a condition of parenchymatous degeneration, secondary to the pneumonia, in *twenty-five cases.*"

In 30 cases there was marked delirum; 15 of the cases were in hard drinkers, and in nearly all a large amount of lung tissue was involved.

In 9 cases there was found more or less meningeal inflammation or congestion.

In 5 cases death occurred with convulsions followed by coma. In 4 of these there existed chronic Bright's disease.

Twelve of the patients died suddenly; 5 of these seemed to die of dyspnœa. "In none of these cases of sudden death did the autopsy show that death was attributable to heart clot."

6. *Trichinosis in Dearborn Co., Ind., in* 1874.

Dr. George Sutton, of Aurora, Ind., in a paper read before the State Medical Society for the current year, presents the histories of ten cases of trichinosis occurring under his observation in 1874. The cases were all propagated by the meat of trichinous hogs, eaten from one table. Three cases resulted in death. There was nothing peculiar in the symptoms of the cases generally, unless it be the uniformity with which severe diarrhœa followed within a few hours the eating of the half-cooked sausages.

Careful post mortem examinations were made in two cases, both in children, with the result of finding very few trichinæ in any of the tissues of the body. There was evidence of severe gastro-intestinal and peritoneal inflammation, however, and from this, the doctor believes the deaths resulted. In these cases there was little or no œdema. In the other case only the muscles of the thigh were examined, and there were found so many trichinæ that it was estimated a cubic inch of muscle contained more than 100,000 of them. There was marked œdema in this case, with soreness of muscles.

The rapid appearance of the parasites in distant parts of the body in so short a time after their development to the migrating size, leads Dr. S. to the conclusion that they are distributed by the circulation.

Extensive observation was made of the flesh of hogs slaughtered in the vicinity, upwards of 1,500 being examined, and trichinæ were found in 3 to 16½ per cent. of them, differing in different lots. Dr. S. believes from his microscopical examinations that the hog cholera is not trichinosis.

7. *A good way to distend the lower bowel in intussusception.*

Dr. Douglas Morton injected into the bowel of a young infant believed to be suffering with intussusception, one-third each of a pair of Seidlitz powders dissolved in an ounce and a half of water, introducing the carbonate solution first. Preventing the escape of the gas, the abdomen was vigorously kneaded. The child was fully anæstetized. Recovery followed.—*Practitioner*, July, 1875.

